# The Role of Myocardial Fibrosis and Bioenergetic Dysfunction in Heart Failure Prognosis: A Study Protocol

**DOI:** 10.7759/cureus.96148

**Published:** 2025-11-05

**Authors:** Mário Barbosa, Ana Melicio, Ana Matias, Mafalda Santos, João M Lopes, Laura Aguiar, Joana Ferreira, Ângela Inácio, Manuel Bicho, Luiz M Falcão

**Affiliations:** 1 Department of Internal Medicine, Hospital Lusíadas Lisboa, Lisbon, PRT; 2 Department of Internal Medicine, Santa Maria Local Health Unit, Lisbon, PRT; 3 Genetics Laboratory, Faculty of Medicine of the University of Lisbon, Lisbon, PRT; 4 Institute of Environmental Health, Associated Earth Laboratory, Faculty of Medicine of the University of Lisbon, Lisbon, PRT; 5 Department of Genetics, Bento da Rocha Cabral Scientific Research Institute, Lisbon, PRT

**Keywords:** bioenergetic dysfunction, galectin-3, gdf-15, heart failure, st2

## Abstract

Introduction

The blockage of the renin-angiotensin-aldosterone and sympathetic nervous systems is fundamental in heart failure (HF) treatment; nonetheless, other mechanisms related to myocardial fibrosis and bioenergetic dysfunction also have a relevant role in the genesis and progression of this syndrome. We aim to stratify the short-term outcome of patients hospitalized with acute decompensated heart failure (ADHF) in New York Heart Association (NYHA) class III or IV based on myocardial fibrosis biomarkers galectin-3 (Gal-3), suppression of tumorigenicity 2 (ST2) and growth differentiation factor15 (GDF-15), and bioenergetic dysfunction markers (absolute iron deficiency and functional iron deficiency). Secondary endpoints were evaluation of short-term prognosis through early HF readmission and all-cause mortality (up to 90 days after hospital discharge), annual heart failure readmission and all-cause mortality, and annual risk for major adverse cardiovascular events (MACE), namely fatal and non-fatal stroke and fatal and non-fatal myocardial infarction, and cardiorenal syndrome.

Material and methods

This cohort prospective observational unicentric study had two arms (left ventricular ejection fraction (LVEF)> 40% and ≤ 40%). A total of 128 participants were included, with 64 per group. Gal-3, ST2, and GDF-15 will be assessed in vitroin serum and plasma, and iron kinetics will be evaluated in plasma. Kaplan-Meier survival estimates will be calculated for each endpoint. A univariate Cox propor­tional hazards model will be performed to achieve hazard ratios and 95% confidence intervals for each variable.

Results

We expect to establish a relation between high values of fibrosis biomarkers, absolute and functional iron deficiency, and impaired short-term outcome in patients admitted due to ADHF.

Conclusions

This study may yield knowledge regarding the role of myocardial fibrosis and bioenergetic dysfunction in HF short-term prognosis.

## Introduction

The growing life expectancy resulting from advancements in healthcare has contributed to an ageing population, which in turn has led to a rise in the incidence of heart failure (HF) [1,2]. HF patients are particularly vulnerable as they have high morbidity and mortality rates, given that this syndrome evolves deleteriously and affects an elderly and feeble population [1]. Despite the decline in its rates, HF remains the leading cause for hospital admissions in both Europe and the United States [3].

HF may have different etiologies that frequently coexist. Diastolic dysfunction may prevail in patients with preserved left ventricular ejection fraction (LVEF), and, on the other hand, overlapping systolic dysfunction may cause reduced ejection fraction. Irrespective of the underlying etiology of HF, LVEF is the undisputed reference used for the assessment of remodeling and, most importantly, to define treatment in light of the available evidence [1].

Regardless of state-of-the-art management in light of the European and American guidelines, diagnostic advances, innovative drugs, and novel therapeutic devices, short-term prognosis, namely readmission and mortality, has failed to improve, leading to a tremendous burden on healthcare systems [1].

The latest European guidelines state that although the prognosis of patients with HF has improved, it remains poor, and the quality of life is also significantly impaired [1]. Adding to this matter, the latest American Guidelines stress a 26% increase in HF hospitalizations and the number of patients admitted due to this condition in the past years [2].

The need for an early detection of risk markers has led to increasing investigations of biomarkers associated with HF physiopathological mechanisms [4]. The current paradigm of HF treatment is centered on the blockage of the renin-angiotensin-aldosterone and sympathetic nervous systems [1,2]; however, other mechanisms linked to myocardial fibrosis and bioenergetic dysfunction also play a relevant role in the genesis and progression of this syndrome. The rationale for investigating the myocardial fibrosis biomarkers Gal-3, ST2, and GDF-15 is that their synthesis results from cardiac injury, namely HF, and may therefore be useful as prognosticators, as they are independent markers of myocardial fibrosis severity and correlate with remodeling and LVEF. Although myocardial fibrosis and bioenergetic dysfunction are physiopathological mechanisms that do not have a direct relation, they share a common hallmark: the prejudicial impact on HF prognosis [4].

This study, Role Of Myocardial Fibrosis And Bioenergetic Dysfunction In Heart Failure Prognosis (ROAD-HF), aims to evaluate if patients with acute decompensated heart failure (ADHF) who present with elevated values of fibrosis biomarkers, absolute and functional iron deficiency, evolve with worse short-term prognosis. In this regard, short-term prognosis was assessed through early HF readmission and all-cause mortality (up to 90 days after hospital discharge), annual HF readmission and all-cause mortality, and annual risk for major adverse cardiovascular events (MACE), namely fatal and non-fatal stroke and fatal and non-fatal myocardial infarction, and cardiorenal syndrome (a third of ADHF patients may develop this complication which aggravates the outcome, as even mild impairments in renal function cause a substantial increase in mortality). Although understanding and recognizing the short-term prognosis of HF holds significant socioeconomic value, national trials focusing on this aspect are limited [5]. Our purpose was to provide insight into the role that myocardial fibrosis and bioenergetic dysfunction play in short-term HF prognosis and raise interest in some promising biomarkers.

## Materials and methods

Study design

This ongoing project started (first participant) on October 25, 2021. It is an observational, prospective cohort, single-center study with two arms: The first group comprises patients with mildly reduced ejection fraction and preserved reduced ejection fraction (LVEF > 40%), and the second group comprises patients with reduced ejection fraction (LVEF ≤ 40% ). We decided to use these two groups, instead of the European Society of Cardiology LVEF classification [1], given that the large majority of HF studies do so, and, since our population study is relatively small, further subgrouping could compromise statistical power.

The study was approved by the Ethics Committee of the Faculty of Medicine of the University of Lisbon Academic Medical Center. The study will be conducted in accordance with its protocol, the Declaration of Helsinki, and the Oviedo Convention. All participants or legal proxies will sign a written informed consent before any study-related activities are initiated.

Study process and data collection

Characterization of the population study will encompass HF-related risk factors (specifically the etiology, the New York Heart Association (NYHA) functional class, LVEF, right ventricular function, clinical signs and symptoms), non-modifiable and modifiable cardiovascular risk factors, associated comorbidities such as chronic kidney disease, anemia, and iron deficiency, as well as biomarkers including N-terminal pro B-type natriuretic peptide (NT-proBNP), high-sensitivity troponin T (hsTnT), and fibrosis biomarkers.

Based on its protocol, patient evaluation includes anamnesis, physical assessment, 12-lead electrocardiogram, postero-anterior thoracic X-ray, blood tests for laboratory analysis, transthoracic Doppler echocardiography, and treatment information.

Each subject will be followed for 12 months in order to assess the annual endpoints (i.e., annual HF readmission and all-cause mortality, and annual risk for fatal and non-fatal stroke and fatal and non-fatal myocardial infarction, and cardiorenal syndrome). For this purpose, follow-up will be conducted using medical records, discharge summaries, death certificates, clinic visits, and telephone consultations.

Endpoints

Our goal is to stratify the short-term prognosis of ADHF patients based on myocardial fibrosis biomarkers (Gal-3, ST2, and GDF-15) and bioenergetic dysfunction markers (absolute iron deficiency and functional iron deficiency). Table 1 summarizes the studied endpoints. 

**Table 1:** Primary and secondary endpoints MACE: major adverse cardiovascular events

Primary endpoint:
1. Relation between Gal-3 and early all-cause mortality (up to 90 days after hospital discharge)
Secondary endpoints:
1. Relation between Gal-3 and:
- early heart failure readmission (up to 90 days after hospital discharge)
- annual heart failure readmission
- annual all-cause mortality
- early heart failure readmission and early all-cause mortality
- annual heart failure readmission and annual all-cause mortality
- annual risk for MACE (namely, fatal and non-fatal stroke and fatal and non-fatal myocardial infarction) and cardiorenal syndrome
2. Relation between ST2, GDF-15, absolute iron deficiency and functional iron deficiency and:
- early heart failure readmission
- early all-cause mortality
- annual heart failure readmission
- annual all-cause mortality
- early heart failure readmission and early all-cause mortality
- annual heart failure readmission and annual all-cause mortality
- annual risk for MACE (namely, fatal and non-fatal stroke and fatal and non-fatal myocardial infarction) and cardiorenal syndrome

Sample size calculation

For the primary endpoint (relation between Gal-3 and early all-cause mortality), the required sample to detect a significant statistical difference with an alpha of 0.05 and a power of 95% is 128 participants, 64 per group, assuming a drop-out/non-compliance/cross-over of 20%. 

Eligibility criteria

Patients are enrolled consecutively from the Department of Internal Medicine II, Santa Maria Hospital in Lisbon, Portugal, according to the criteria depicted in Table 2 and Table 3.

**Table 2:** Inclusion Criteria NYHA: New York Heart Association; ADHF: acute decompensated heart failure

Charateristics
Hospitalization due to ADHF in class III or IV of NYHA
Age > 18 years old

**Table 3:** Exclusion criteria

Characteristics
In-hospital death in the first admission
Hospital discharge against clinical advice
Chronic kidney disease patients with an estimated glomerular filtration rate< 30 mL/min/1.73 m² (calculated with the Modification of Diet in Renal Disease score) or under renal replacement therapy
Moderate or severe hepatic impairment (calculated with the Child-Pugh score)
Active neoplasm with or without metastasis

Masking

It is a double-blind study, given that patients ignore the results of the biomarkers, and investigators will only acknowledge the measurements of the biomarkers at study completion.

Definitions

The diagnosis of HF, as the diagnosis of absolute and functional iron deficiency associated with HF, will follow the European Society of Cardiology (ESC) guidelines [1]; treatment, including intravenous ferric carboxymaltose, will be optimized according to the same guidelines [1].

Biomarkers dosing

Myocardial fibrosis and bioenergetic dysfunction biomarkers will be collected after clinical stabilization. Bearing in mind that the evolution of fibrosis biomarkers with the progression and decompensation of HF is unclear, we standardized biomarkers’ sampling after clinical stabilization as defined in large-scale studies addressing patients hospitalized for AHF. Hemodynamic stability is defined by maintenance of a systolic blood pressure ≥100 mmHg for the previous six hours, with no increase in the dose of intravenous diuretics and no need for intravenous vasodilators during the preceding six hours, and no use of intravenous inotropes in the last 24 hours [6,7].

Biomarkers will be measured in vitro using plasma and serum samples, which will be stored at the study sites at -20ºC, followed by storage at -80ºC. Gal-3 and ST2 will be measured using pre-coated human ELISA kits (R&D Systems, Inc., Minneapolis, Minnesota, United States) while GDF-15, NT-proBNP, and hs-TnT values will be assessed with electroche­miluminescence immunoassay (Elecsys®; Roche Holding AG, Basel, Switzerland).

Renal, hepatic, and thyroid function, along with ionogram and iron kinetics, will be determined using the same sample, and haematological values will be analyzed using ethylenediaminetetraacetic acid whole blood.

In order to reduce measurement error and increase reproducibility, standardized procedures, as defined by the manufacturers, and calibrated instruments will be used by experts.

Statistical analysis

Descriptive Analysis

Continuous variables will be analyzed by mean, median, standard deviation (SD), interquartile range, and range. The comparison amongst participants will be made for all variables using the t-test or the Mann-Whitney U test. Categorical variables will be defined by relative and absolute frequencies and matched using the chi-squared test or Fisher’s Exact test as appropriate. The Shapiro-Wilk test will be applied to assess the normality of continuous variables.

Analysis of Endpoints

A survival analysis will be performed for each event of interest (i.e., early rehospitalization, early mortality, annual rehospitalization, annual mortality, and annual MACE and cardiorenal syndrome). Kaplan Meier survival estimates will be computed for each categorical variable corresponding to each outcome. The log-rank test will be applied to assess differences in survival probabilities among the variables. A univariate Cox proportional hazards model will be used to determine HR and 95% confidence intervals. The proportional hazards assumption will be addressed through Schoenfeld residuals.

The results of the study will be divulged in scientific meetings and published in scientific peer-reviewed journals.

## Results

Based on a previous study with a similar design performed by the authors [4], we expect to establish a relation between elevated values of fibrosis biomarkers, absolute and functional iron deficiency and impaired short-term outcome in patients admitted due to ADHF.

## Discussion

HF is the leading cause of hospitalization in Europe and in the United States of America, accounting for around one million admissions annually [5]. Based on the most recent European guidelines, HF is estimated to affect 1-2% of the adult population [1]; moreover, the American Heart Association stated that the number of HF patients will double until 2030, affecting more than eight million people in the United States (one in every 33), and consequently, its social burden [6].

Although large-scale registries have documented a decline in hospitalization due to HF, the rehospitalization rate remains high [7] since patients admitted for ADHF have a substantial risk of readmission and death, particularly in the first 90 days post discharge [8]. Around a quarter of the patients are readmitted in the first 30 days post discharge, and approximately one-third are rehospitalized in the first 90 days post discharge [6]. Strikingly, mortality in the first 90 days post discharge may reach a rate of 15% [9].

The first 30 to 90 days post discharge are a critical period for HF patients and should be an opportunity to intervene and try to change the syndrome’s course. Since it is not viable to reevaluate all the HF hospitalized patients in this strict period of time, it is mandatory to identify and target high-risk patients for early readmission and mortality, and be assertive in this vulnerable period.

Regarding Portugal, the EPICA (Epidemiology of Heart Failure and Learning) study in 2002 was the only national prevalence trial for more than a decade [10]. Data from the national census estimate that there are around 380,000 patients suffering from HF in Portugal [11], and it is expected that it could increase to an overwhelming 500,000 patients in 2035 [12]. Between 2004 and 2012, HF hospitalizations increased 33% in Portugal, as for the number of readmissions, namely in the first 30 days post discharge (14.6%) [13].

According to the PORTHOS (Portuguese Heart Failure Prevalence Observational Study) study, which was released in October 2023, one in six adults over 50 years of age suffers from HF in Portugal [14]. Data from 2014 revealed that HF had an impact of around 400 million euros, and it was anticipated that this could reach an astronomical amount of 500 million euros in 2036. Notably, the main cause of this expenditure is related to admissions [15].

Given its growing prevalence and socio-economic burden, allied to its poor prognosis, it is of utmost importance to stratify the risk of HF patients, aiming to improve short-term readmission and death prognosis.

The need to dispose of risk markers that detect early phases of the disease in order to abrogate its inexorable evolution has led to the study of different pathophysiological pathways, specifically those related to myocardial fibrosis and bioenergetic dysfunction. Myocardial fibrosis causes ventricular malfunction, favoring HF progression [16].

The synthesis of Gal-3 by activated macrophages stimulates fibroblast and collagen proliferation in the myocardium. The interest in such a biomarker is based on the evidence that its expression is negligible in individuals without HF and augments rapidly as HF evolves, emerging as an independent marker of myocardial fibrosis severity [17]. Remodeling, fibrosis, and myocardial hypertrophy trigger the production of ST2, which seems to play a cardioprotective role as it inhibits the noxious effect of fibrosis, improving cardiac function [18]. Its relevance is substantiated by the fact that ST2 is a marker of cardiac remodeling, which correlates with LVEF [19]. The former recommendation for Gal-3 and ST2 as complementary HF prognosticators by the American College of Cardiology and the American Heart Association was dropped in the latest actualization [20]. Regarding the European guidelines [1], the measurement of these biomarkers for additive risk stratification has never been supported. Another biomarker to take into consideration is GDF-15, which is a stress cytokine released in response to cardiac insult, like HF. It is important to explore this promising biomarker since it promotes cardiac remodeling through fibrosis induction and is linked to LVEF [21].

Myocardial fibrosis biomarkers arise as a supplement to the clinical routine as they may identify at-risk HF patients who may require zealous follow-up. In the near future, the blockade of myocardial fibrosis pathways may emerge as a novel complementary therapeutic target; therefore, it is fundamental to acknowledge the independent role that each biomarker plays in HF prognosis.

Regarding bioenergetic dysfunction, it is known that iron deficiency leads to myocardial contractility impairment due to mitochondrial malfunction, which compromises the production of energy related to HF. The current HF therapeutic paradigm is centered on the reduction of energetic consumption, neglecting the energetic dysfunction inherent to this syndrome. HF increases myocardial energy demand and compromises, concomitantly, the energetic metabolism [22]. It seems that HF leads to myocardial iron impairment; meanwhile, myocardial iron scarcity exacerbates HF, in a vicious cycle [23].

Iron deficiency has an estimated prevalence of 50-80% in ADHF [24]. Bearing in mind this unbalance in energetic homeostasis, HF can be considered a bioenergetic disease [22] in which iron deficiency is a marker of bioenergetic dysfunction [25]. The European guidelines [1] recommend iron correction in absolute and functional iron deficiency, aiming to improve symptoms, functional capacity, and quality of life and to reduce hospitalizations in light of the available scientific evidence [26].

Despite current scientific data pointing Gal-3 as a prognosticator of mortality, there is some controversy concerning its utility in predicting rehospitalization risk [27]; hence, addressing its impact in HF rehospitalization could yield knowledge to this theme. The main studies regarding the prognostic value of ST2 in HF [28] overlooked 90-day readmission; therefore, the analysis of this endpoint could complement the current knowledge. With regard to GDF-15, we believe that its investigation could be profitable since it has not been validated yet by the main Societies [1,20] and given the fact that it has been assessed, essentially, in chronic HF, leading to scarce evidence in ADHF [21]. The most recent studies have related iron deficiency to rehospitalization risk in ADHF (AFFIRM-AHF) [29] and in ambulatory HF patients (IRONMAN) [30]. The assessment of hard endpoints, namely mortality, may further add information to this subject.

Study limitations

Our study protocol has limitations that should be taken into consideration. The fact that it was a single-center study may hinder the generalization of the findings; thus, validation through larger, multicentric studies will be necessary before widespread application. Furthermore, the study population being considered is not very large, requiring adequate statistical analysis to ensure the credibility of the results. Regarding biomarkers, some thresholds are not standardized, and dissimilar techniques of quantifying some biomarkers are not directly comparable, which has to be taken into account when confronted with other investigations. Despite these potential limitations, this research discloses data from a real-world clinical context, and the findings can be compared with national data and prior large-scale trials.

## Conclusions

Through this study, we expect to establish a relation between high values of fibrosis biomarkers, absolute and functional iron deficiency, and impaired short-term outcome in patients admitted due to ADHF. This study could add information to the role that promising fibrosis biomarkers and bioenergetic dysfunction may play in the short-term prognosis of HF.
